# Quantitative Fit Testing on Filtering Facepiece Respirators in Use by Peruvian Healthcare Workers Caring for Tuberculosis Patients during the COVID-19 Pandemic: PROFIT Study 2020

**DOI:** 10.3390/ijerph20166618

**Published:** 2023-08-21

**Authors:** Jorge Inolopú, Kevin Mayma, Maricela Curisinche-Rojas, Rula Aylas, Juan A. Flores, Jaime Rosales-Rimache

**Affiliations:** 1 Centro Nacional de Salud Ocupacional y Protección del Ambiente para la Salud, Instituto Nacional de Salud (CENSOPAS), Lima 15046, Peru; jinolopu@ins.gob.pe (J.I.); kmayma@ins.gob.pe (K.M.); jrosales@ins.gob.pe (J.R.-R.); 2 Centro Nacional de Salud Pública, Instituto Nacional de Salud, Lima 15066, Peru; mcurisinche@ins.gob.pe; 3 Dirección de Prevención y Control de Tuberculosis, Ministerio de Salud, Lima 15072, Peru; aylas_rula@yahoo.com; 4 Escuela Profesional de Tecnología Médica, Universidad Privada San Juan Bautista, Lima 15067, Peru; 5 Instituto de Investigación en Salud Global, Universidad Privada San Juan Bautista, Lima 15067, Peru

**Keywords:** respirator, fit testing, tuberculosis, respiratory protection

## Abstract

Background: The COVID-19 pandemic has promoted a shortage of filtering facepiece respirators (FFRs) and the emergence of new FFRs brands. We aimed to determine the fit provided by in-use FFRs in Peruvian healthcare workers (HCWs) during the COVID-19 pandemic. Methods: We enrolled 279 HCWs from 37 primary healthcare centers with highest burden of care for TB in Peru, of which 263 were assessed using quantitative fit tests (QNFT). Results were expressed as real-time fit factor (rt-FF) and overall fit factor (overall-FF), which was categorized as ≥100 (optimal result), 50–99, and <50. Results: We identified 3M 1860 FFRs (33.1%), Xiantao Zhong Yi ZYB-11 FFRs (24.6%) and Makrite 9500 FFRs (20.5%), mainly. Eighty-seven FFRs (33.1%) had an optimal overall-FF, 27 (10.3%) between 50–99, and 149 (56.6%) less than 50. Of the 87 FFRs with optimal overall-FF, 73 (83.9%) were 3M 1860 FFRs. Of the 27 FFRs with overall-FF between 50–99, 7 (25.9%) were Makrite 9500, while of the 149 with overall-FF less than 50, 58 (38.9%), and 47 (31.5%) were Xiantao Zhong Yi ZYB-11 and Makrite 9500, respectively. Conclusion: Xiantao Zhong Yi and Makrite FFRs do not adapt adequately to the face of Peruvian HCWs, most having fit factors less than 50.

## 1. Introduction

Traditionally, tuberculosis (TB) has been the leading occupational health problem in healthcare workers (HCWs) who provide medical care under poor infection control conditions [1]. However, during 2020 and 2021, the disease caused by SARS-CoV-2 (COVID-19) has become the primary occupational respiratory infection in unvaccinated HCWs [2]. The World Health Organization (WHO) recommends the use of filtering facepiece respirators (FFRs) of the American N95 certification (NIOSH 42 CFR 84) and the European FFP2 or FFP3 certification (EN149:2001) for the care of patients with TB and COVID-19, particularly in situations involving aerosol-generating procedures [3,4,5]. Type N95 and FFP2 FFRs provide 94–95% filtration efficiency of aerosol particles with a mean diameter of 0.3 µm [6]. 

However, the protective capacity of FFRs is not only based on filtration efficiency, it is crucial to achieve an optimal facial fit [7]. A non-optimal facial fit can account for one-sixth of the airflow entering the FFRs [8], one of the main routes of contamination [9]. The assessment of facial fit is named the fit test and is the main parameter of the Respiratory Protection Program proposed by the National Institute for Occupational Safety and Health (NIOSH) [10]. According to the US Occupational Safety and Health Administration (OSHA), the fit test should be administered to HCWs to determine the FFR (brand, model, and size) that provides them an optimal fit according to their facial dimensions and to ensure its continuous replacement by the employer [11]. There are two types of fit tests: qualitative (QLFT) and quantitative fit test (QNFT). QLFT assesses fit qualitatively by detecting substances with distinct odor and flavor that pass through the edges of the FFR (presence or absence of fit). In contrast, QNFT is an analytical procedure that quantifies the level of fit of the FFR known as the fit factor [12,13].

The fit test is an essential procedure in the field of occupational health, specifically aimed at HCWs exposed to TB in endemic countries [14]. In this regard, Peru is the country with the second highest burden of TB in Latin America and the Caribbean, [15] and one of the 30 countries with the highest burden of Multidrug-Resistant TB (MDR-TB) in the world [16]. Peruvian legislation highlight the importance of administrative, environmental, and respiratory protection control measures for the prevention and control of TB [17]. Nevertheless, fit testing among Peruvian HCWs is largely absent due to the lack of a respiratory protection program [18]. Additionally, due to the global shortage of FFRs generated by the COVID-19 pandemic, new FFRs with poorly technical specifications were provided to HCWs [19]. In this sense, we applied QNFT in FFRs worn by HCWs who care for TB patients under our PROFIT (PROmoting the FIT) study 2020.

## 2. Materials and Methods

### 2.1. Study Design

The PROFIT study 2020 was conducted by the National Center for Occupational Health and Environmental Health (CENSOPAS in Spanish) of the Peruvian National Institute of Health as part of its Institutional Operational Plan. During November and December 2020, we visited 37 primary health care centers (PHCs) with the highest number of TB case care in the metropolitan area of Lima and Callao, Peru, according to data from the SIGTB platform (TB management information system) of the Tuberculosis Prevention and Control Directorate of the Ministry of Health of Peru [20]. Our aim was to enroll HCWs who were under working conditions, in order to apply QNFT to the FFRs they were using. The enrollment was for convenience according to the time availability of HCWs, prioritizing those who work in the TB Control Program, the unit responsible for detecting, diagnosing, and treating TB cases in PHC [21]. We collected data from enrolled HCWs regarding their gender, occupation, age, work area, and the time of use of FFR using a questionnaire. The FFRs evaluated by QNFT were N95, FFP2, KN95, and other equivalent types. They were characterized according to their brand, model, type, size, design, presence of batch number on their surface, and country of origin of the manufacturer. We excluded from the analysis HCWs with facial hair [22], with 3M 1860 FFRs suspected of counterfeiting [23], or with expired FFRs as long as we had access to the boxes where the expiration date is indicated.

### 2.2. Quantitative Fit Testing

We applied QNFT based on condensation nuclei count using the model 8048 PortaCount^®^ instrument (TSI Inc., St. Paul, MN, USA) that measures ambient aerosol particles in the 0.02 to 1 micron size range [24,25]. We determined two fit factors: real-time fit factor (rt-FF) and overall fit factor (overall-FF) before a daily pass check (particle check, zero check, maximum fit factor test, and ambient concentration check) according to the manufacturer’s instructions. Initially, we determine the rt-FF to train on properly donning the FFRs and making adjustments in real-time [24]. The rt-FF is updated with intervals per second on the PortaCount^®^ screen when using FitCheck^®^ mode and allows us to identify non-tight adjustment areas to adapt the FFRs to face manually. Subsequently, we determine the overall-FF by applying the OSHA 29 CFR 1910.134 Appendix A standard, which consists of eight movements that simulate work conditions: normal breathing, deep breathing, left-right head turns, up-down head turns, speaking loudly, gestures and grimaces, leaning forward and normal breathing [26]. Each movement lasted 60 s except for gestures and faces, which lasted 15 s. A fit factor (rt-FF or overall-FF) equal to or greater than 100 was optimal (passing result), while a fit factor less than 100 was non-optimal (failed result). The QNFT were carried out in environments without a high air flow and at an ambient temperature that fluctuated between 22 and 25 °C according to the summer season. The environmental conditions were favorable, and we did not need particle generating equipment.

### 2.3. Procedures

The HCWs were requested to provide their FFRs, which were then connected to the PortaCount^®^ through an air sampling line. Afterward, we ask them to put on the FFRs as they usually do at work without our support and 10 s later the pre-instruction rt-FF was recorded. The post-instruction rt-FF was recorded 10 s after providing instruction focused on improving the position of the elastic band, adjusting the nose clip, and fit check based on the recommendations of the Centers for Disease Control and Prevention [27]. The results of rt-FF pre- and post-instruction were determined only for the last 184 (70.3%) HCWs because this process was incorporated during the study. Regardless of the result of the rt-FF, we subsequently determined the overall-FF. We verified that no HCWs presented respiratory symptoms to perform the QNFT. However, each PortaCount^®^ sample line was cleaned with isopropyl alcohol at the end of each QNFT for all HCWs, in accordance with the manufacturer’s recommendations in the context of the COVID-19 pandemic.

### 2.4. Data Analysis

We performed an descriptive analysis to express the fit factors (rt-FF or overall-FF) as geometric means with a 95% confidence interval and were also categorized into two groups: ≥100 and <100. The <100 category was arbitrarily subcategorized into 50–99 and <50 for a better understanding of the level of fit. Likewise, we performed a bivariate analysis to relate sex, age, design of the FFRs (foldable or conical) with the fit factors obtained. Consequently, the pre- and post-instruction rt-FF categories were compared using a Pearson chi-squared test with a significance of 95%. In addition, we evaluated Spearman’s rank correlation coefficient to compare hours of FFRs use and overall-FF by type of FFRs. The data management and analysis were performed using Stata software version 16 (Stata Corporation, College Station, TX, USA).

## 3. Results

### 3.1. Description

We interviewed 279 HCWs of 37 PHC of metropolitan area of Lima and Callao in Peru of them we enrolled 263 (94.6%) of different working areas (Table S1). The coverage of QNFT provided to the HCWs of the TB Control Program was 52.4% considering that we were able to enroll 89 of a total of 170 HCWs from that work area in the 37 PHCs during the study. Regarding the 16 excluded HCWs, the reasons were the following: 11 had facial hair, 3 had counterfeit FFR, and 2 had expired FFRs. However, due to the characteristics of the PROFIT study 2020, the excluded HCWs were also evaluated by QNFT to provide evidence to health authorities (Table S2). Of the 263 HCWs enrolled, we obtained rt-FF pre- and post-instruction results in 184 (70.3%) and overall-FF results in all the 263 HCWs (Table S3).

**Figure 1:**
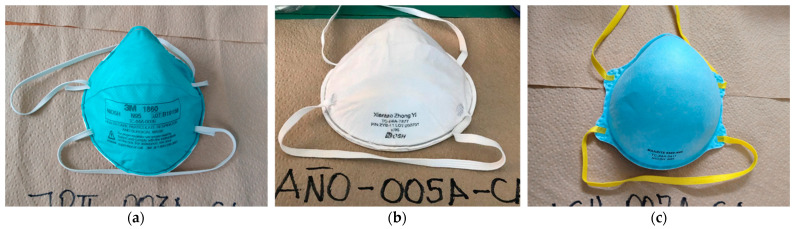
Top three FFRs identified in the study: (**a**) 3M 1860; (**b**) Makrite 9500; (**c**) Xiantao Zhong Yi ZYB-11.

We identified 12 FFRs models which were all standard size and predominantly of Asian origin: Chinese (91/263) and Taiwanese (55/263) (Table 1 and Figure 1). All the FFRs were provided by the PHC except 3 FFRs, which the HCWs acquired on their account: 2 3M 1860 FFRs and 1 Lucca Light FFR. The main FFRs identified were 3M 1860 (33.1%), Xiantao Zhong Yi ZYB-11 (24.7%), and Makrite 9500 (20.5%). All the Xiantao Zhong Yi ZYB-11 FFRs identified in the 37 PHCs belonged to only 3 batch numbers (L200-701; L200-601; and L200-901), while the 3M 1860 FFRs had 18 different lot numbers. The Makrite, Grande, Y&Z, PGT Care, Lucca Light, and Giko FFRs evaluated in our study do not display their batch numbers on their surface.

### 3.2. Instruction and Real-Time Fit Factor Assessment

The proportion of optimal rt-FF category increased significantly from 11.4% to 39.1% (*p* < 0.01), while the proportion of non-optimal rt-FF category less than 50 decreased significantly from 79.4% to 45.1% (*p* < 0.01) (Table 2). The 3M 1860 FFRs had an optimal pre-instructional rt-FF of 38.3% (18/47) and a significantly higher optimal post-instructional rt-FF of 93.6% (44/47) (*p* < 0.01). The 3M 9010 FFRs had an optimal pre-instruction rt-FF of 5.6% (1/18) that increased significantly post-instruction to 61.1% (11/18) (*p* < 0.01). Similarly, 3M 9920H FFRs had an optimal pre-instruction rt-FF of 9.1% (1/11), increasing to 54.5% (6/11) (*p* = 0.01). Neither Xiantao Zhong Yi ZYB-11 nor Makrite 9500 FFRs achieved optimal pre-instruction rt-FF, but after instruction, five of them achieved an optimal rt-FF. Grande and PGT FFRs showed minimal increase in optimal rt-FF ratio, while KN95, Y&Z, Lucca Light, and Giko FFRs did not show optimal post-instruction rt-FF.

### 3.3. Overall Fit Factor Assessment

The 3M 1860 showed an optimal overall-FF at 73 (83.9%) FFRs. The 3M 9010 and 3M 9920H FFRs together had an optimal overall-FF of 33.4% (10/30). The Xiantao Zhong Yi ZYB-11 FFRs obtained a single optimal overall-FF result (1.5%), while the Makrite 9500 Lucca Light, Giko, and Benehal FFRs did not get optimal overall-FF result. The 56.7% of FFRs evaluated had an overall-FF less than 50 and were represented mainly by two of the most widely used FFRs: Xiantao Zhong Yi ZYB-11 (58/149) and Makrite 9500 (47/149)**.** It should be noted that 3M FFRs (1860 and 9010 models) were present mainly in the HCWs of the TB Control Program (49/89).

Of the 263 evaluations carried out, 87 (33.1%) HCWs had an optimal overall-FF, 27 (10.3%) had an overall-FF between 50–99, and 149 (56.6%) had an overall-FF less than 50 (Table 3). Of the 87 HCWs with optimal overall-FF, 73 (83.9%) were 3M 1860 FFRs. The 3M 1860 FFRs had a geometric mean of 126.3 (95% CI: 109.4–146.6), the only one that exceeds the threshold of 100. Likewise, of the 27 HCWs with overall-FF between 50–99, 7 (25.9%) were Makrite 9500 FFR, while of the 149 with overall-FF less than 50, 58 (38.9%) and 47 (31.5%) were Xiantao Zhong Yi ZYB-11 and Makrite 9500, respectively. The FFRs assessed in the QNFT had a median usage time of 12 h (interquartile range: 18.0) and did not correlate with the overall-FF (*p* > 0.05). Likewise, regarding the three main FFRs (3M 1860, Xiantao Zhong Yi ZYB-11, and Makrite 9500), our bivariate analysis showed that gender, and age were not related to overall FF (*p* > 0.05).

**Figure 2:**
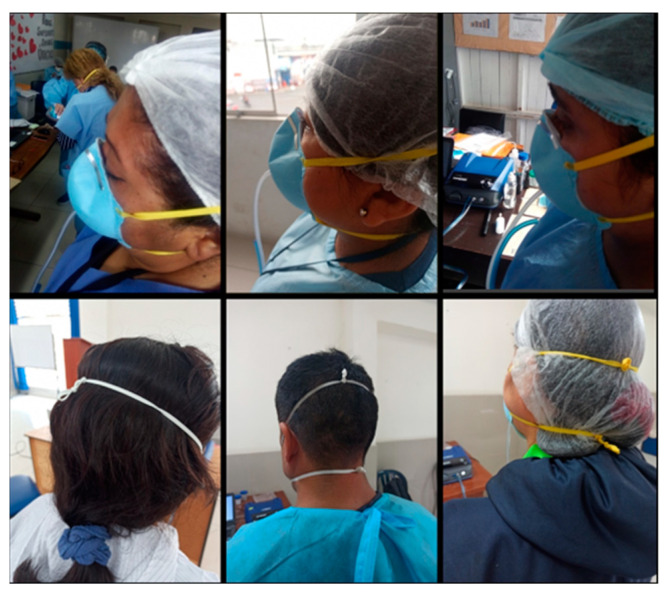
Visible openings in the nasal area in HCWs using the Makrite 9500 FFRs (**top** photographs) and loss of FFRs adjustment mainly in users with elastic bands that are too extended and therefore must be tied to increase the FFRs adjustment (**lower** photographs).

It is important to mention that during the evaluation we noticed that the nose clip of most of the Makrite and Xiantao Zhong Yi ZYB-11 FFRs did not adapt correctly to the shape of the nose of the HCWs. Despite the attempts to adjust the FFRs during the training, noticeable openings formed in the nasal area that would explain why we obtained very low levels of rt-FF and overall-FF. Some HCWs reported that the nose clip of Makrite FFR, initially in the form of an arch, has limited malleability and yields to its original shape after adjustment. We also observed a loss of fit in the chin region, partly evidenced by the excess length of the elastic bands. Given this, some HCWs reported that the elastic bands tend to remain stretched due to the loss of their elasticity, and therefore, they must tie them to increase the fit (Figure 2).

## 4. Discussion

Our study revealed a non-optimal overall-FF of 67.0% (176/263) of the evaluated HCWs, of which 84.6% (149/176) present an overall-FF of less than 50 and it was not related to sex, age, design of the FFRs or time of use of the FFR. The 3M 1860 FFRs had a significantly higher adaptability capacity for facial adjustment than the Xiantao Zhong Yi ZYB-11 and Makrite 9500 FFRs. Indeed, the Xiantao Zhong Yi ZYB-11 and Makrite 9500 FFRs showed poor adaptability to the facial dimensions, mainly in the nasal and chin areas. The lack of adaptability of the FFRs in our study may be mainly due to 3 factors: (i) incorrect size, (ii) damage to its support structure (elastic bands, nose clip) due to excessive reuse, and/or (iii) deficiencies in its filtration efficiency.

First, all FFRs were standard size because the purchase specifications do not take into account the size of the FFRs. The HCWs of the Peruvian public sector do not have the power to choose the brand, model or size of the FFR, promoting that they acquire them on their own. Second, in our context FFRs are widely reused and it is not clear for how long they can be used. Added to this, there is no comprehensive educational program based on workshops aimed at HCWs on the use of FFRs, which could favor their excessive reuse and deterioration that significantly affects their adjustment. Evidence shows that the noses clip and the elastic bands of the FFRs can show serious flaws and affect the facial fit, being necessary to be evaluated more precisely with appropriate instruments [28]. Third, we cannot rule out that there are FFRs with deficiencies in their filtration capacity as a result of their deterioration, expiration or adulteration. Given this, there are some local initiatives that could be incorporated into the testing of the FFRs [29].

On the other hand, although in small quantity, we identified FFRs that showed insufficient technical characteristics. Six FFRs only display the KN95 GB2626-2006 inscription on their surface, with no known brand, model, or manufacturer. The Y&Z FFRs identified in our study was produced by the Fido Mask Company and had a NIOSH TC-841-4227 voluntary certification revocation since August 2014 [30]. It cannot be manufactured, assembled, sold, or distributed as a NIOSH-approved product, so it is probably expired. We also identified a Lucca Light FFRs of Chinese origin and an unknown manufacturer that was banned in the European Union for having a filtration efficiency of less than 57.2% [31]. The Y&Z FFRs was worn by a nursing staff and provided by the PHC, while the Lucca Light FFRs was worn by a security staff and purchased at their own expense.

Except for 3M FFRs, there is no information in the literature on fit testing in most of the FFRs evaluated in our study. A recent study evaluated the overall-FF of 03 FFRs: Xiantao Zhong Yi ZYB-11, Makrite 9500, and KN95 (manufactured by Zhong Jian Le of Chengde Technology Co., Ltd., Wenzhou, China) in 07 HCWs [32]. Of the 07 HCWs with the Xiantao Zhong Yi FFR, none had an optimal overall-FF; however, all had values less than 60. On the other hand, of the 7 HCWs with the Makrite FFR, one had an optimal overall-FF; however, the rest had an overall-FF lower than 20. Regarding the KN95 FFRs, the overall-FF results were all lower than 3, having the same performance as a cloth mask. The study suggests that the fit loss was due to the inadequate sealing of the FFRs in the chin area.

One publication evaluating the tightness of FFRs available at a PHC in the United States found KN95 FFRs that did not display brand, model, or lot number data; and had non-optimal overall-FF results [33]. These FFRs did not show elastic band support on the head and had perforations on their surface with the text “KN95” and “GB2626-2006” in low-relief that gave rise to a thin non-protective layer of the filter. Likewise, a study applied a qualitative fit test to a panel of 7 individuals using 12 types of FFRs between KN95 and N95 FFRs (3M 1860) and their results showed an optimal fit percentage of 3% (1/36) for KN95 FFR, while the 3M 1860 FFRs had a 100% (12/12) best fit [34].

Our study has several limitations. The enrollment was conducted in a small sample obtained for convenience from a non-representative group of HCWs from 37 PHCs in metropolitan Lima and Callao. The low coverage achieved in TB Control Program staff was mainly due to the absence of some HCWs during our visit due to remote work. However, it should be noted that no worker interviewed refused to participate in the study. Regarding the analysis, we applied a single overall-FF measure per FFRs in use, unlike other studies that perform up to three measurements for precision purposes [35,36]. Likewise, we did not carry out a multivariate analysis that allows us to explore other interpretations of the overall-FF due to the small sample obtained and that the study was not planned during study conception. On the other hand, we did not check the expiration date of all the FFRs because that depended on access to the boxes. We were able to identify counterfeit 3M 1860 FFRs since we have clear specifications of their original characteristics. However, we could not identify counterfeit FFRs from other brands, such as the Makrite 9500. This point is essential given the continual reporting of fake alerts by Makrite displayed on their website [37]. Therefore, our results should be taken with caution.

The PROFIT study allowed us to evaluate respiratory protection measures focused on FFRs facial fit in Peruvian HCWs in the context of the COVID-19 pandemic. The main strength of the study was the application of QNFT, which does not depend on the sensitivity or attitude of the person, unlike qualitative fit tests [38]. In addition, the QNFT based on condensation nuclei counting that we use, unlike the QNFT based on controlled negative pressure, is not affected by the breathing or movements of the study subject. We believe that our findings are important for countries with endemic TB that have not yet implemented respiratory protection measures. Finally, it is essential that the PHC, through the Occupational Health and Safety services, comply with the programs for the prevention and control of occupational risks and diseases, including those transmitted by air, to strengthen the primary prevention activities in workplaces.

## 5. Conclusions

We found a non-optimal fit provided by in-use FFRs in HCWs during the COVID-19 pandemic. Most of these FFRs were new brands not previously used by HCWs, which appeared due to shortages of FFRs. This finding helps us to understand the exposure to TB transmission during a pandemic scenario where there was a limited access to diagnosis, follow-up and treatment that could increase the TB cases in settings with precarious health system. We recommend implementing measures that prioritize educational interventions with a practical approach to recognizing the FFR, correct handling, fit check, and good storage practices. Compliance with these activities must be monitored, supervised, and evaluated by the Respiratory Infection Control Plan established in the Technical Health Standard for TB Care in Peru.

## Figures and Tables

**Table 1:** Characteristics of the FFRs in use by HCWs in the study.

Brand, Model, and Type of FFRs	NIOSH Approval Number	Design of FFR	Batch Number on FFR	Manufacturer	PHC ^a^	*n* (%)
3M, 1860, N95	TC-84A-0006	Cup/headloop	Yes	3M, Maplewood, MN, USA	19	87 (33.1)
Xiantao Zhong Yi, ZYB-11, N95	TC-84A-7877	Cup/head loop	Yes	Xiantao Zhongyi Safety Protecting Products Co., Ltd., Xiantao, China	21	65 (24.6)
Makrite, 9500, N95	TC-84A-5411	Cup/headloop	No	Makrite Industries Inc., Taipei, Taiwan	17	54 (20.5)
3M, 9010, N95	TC-84A-4243	Folding/headloop	Yes	3M, Maplewood, MN, USA	12	19 (7.2)
3M, 9920H, PFF	-	Folding/head loop	Yes	3M, Sao Paulo, Brasil	3	11 (4.2)
Grande, CDN3S-P2, FFP2	-	Cup/head loop	No	Jiangyin Chang-hung Industrial, JiangYin, China	8	11 (4.2)
PGT Care, PGT-0095, FFP2	-	Folding/earloop	No	Fujian Dahong Industry & Development Co., Ltd., Shishi, China	3	6 (2.3)
Brand and model unknown, KN95 ^b^	-	Folding/earloop	No	Unknown, China	5	6 (2.3)
Giko, 1200H, N95	TC-84A-4006	Cup/head loop	No	Shanghai Gangkai Purifying Products Co., Ltd., Shanghai, China	1	1 (0.4)
Benehal, MS6115L, N95	TC-84A-8474	Cup/head loop	Yes	Suzhou Sanical Protective Product Manufacturing Co., Ltd., Suzhou, China	1	1 (0.4)
Y&Z, Safety Work F720, N95	TC-84A-4227	Cup/head loop	No	Fido Mask Co., Ltd., Taichung, Taiwan	1	1 (0.4)
Lucca Light, Lucca Care, KN95/FFP2	-	Folding/earloop	No	Unknown, China	1	1 (0.4)
Total						263

^a^ Number of primary healthcare center where the FFRs were found of the 37 evaluated centers. ^b^ FFRs with no brand or model present on its surface.

**Table 2:** Real-time fit factors in evaluated FFRs in use by HCWs in the study.

Brand, Model, and Type of FFRs	rt-FF Pre-Instruction, *n* = 184				rt-FF Post-Instruction, *n* = 184				*n* (%)
	<50	50–99	≥100	Geometric Mean (IC 95%)	<50	50–99	≥100	Geometric Mean (IC 95%)	
	*n* (%)	*n* (%)	*n* (%)		*n* (%)	*n* (%)	*n* (%)		
3M, 1860, N95	19 (40.4)	10 (21.3)	18 (38.3)	42.2 (27.5–64.5)	1 (2.1)	2 (4.3)	44 (93.6)	168.7 (141.4–201.5)	47 (25.5)
Xiantao Zhong Yi, ZYB-11, N95	34 (94.4)	2 (5.6)	-	8.0 (5.6–11.3)	25 (69.4)	10 (27.8)	1 (2.8)	29.0 (21.0–40.0)	36 (19.6)
Makrite, 9500, N95	46 (97.9)	1 (2.1)	-	4.0 (2.9–5.6)	33 (70.2)	10 (21.3)	4 (8.5)	24.6 (18.1–33.4)	47 (25.5)
3M, 9010, N95	14 (77.8)	3 (16.7)	1 (5.6)	7.22 (3.1–16.9)	3 (16.7)	4 (22.2)	11 (61.1)	98.0 (61.1–157.2)	18 (9.8)
3M, 9920H, PFF	9 (81.8)	1 (9.1)	1 (9.1)	12.7 (6.2–26.1)	4 (36.4)	1 (9.1)	6 (54.5)	62.3 (27.3–142.2)	11 (6.0)
Grande, CDN3S-P2, FFP2	10 (90.9)	-	1 (9.1)	6.5 (2.5–16.8)	6 (54.5)	2 (18.2)	3 (27.3)	22.8 (6.8–76.5)	11 (6.0)
PGT Care, PGT-0095, FFP2	6 (100)	-	-	7.6 (2.2–25.7)	3 (50.0)	-	3 (50.0)	53.5 (13.0–220.8)	6 (3.3)
Brand and model unknown, KN95 ^a^	5 (100)	-	-	2.6 (0.7–9.3)	5 (100)	-	-	13.7 (4.4–42.2)	5 (2.7)
Giko, 1200H, N95	1 (100)	-	-	-	1 (100)	-	-	-	1 (0.5)
Y&Z, Safety Work F720, N95	1 (100)	-	-	-	1 (100)	-	-	-	1 (0.5)
Lucca Light, Lucca Care, KN95/FFP2	1 (100)	-	-	-	1 (100)	-	-	-	1 (0.5)
Total	146 (79.4)	17 (9.2)	21 (11.4)	9.7 (7.8–12.2)	83 (45.1)	29 (15.8)	72 (39.1)	49.1 (40.5–59.6)	184

^a^ FFRs with no brand or model present on its surface.

**Table 3:** Overall fit factors in evaluated FFRs in use by HCWs in the study.

Brand, Model, and Type of FFRs	Hours of FFRs Use ^a^	Overall-FF, *n* = 263				*n* (%)
		<50	50–99	≥100	Geometric Mean (IC 95%)	
		*n* (%)	*n* (%)	*n* (%)		
3M, 1860, N95	12 (19)	9 (10.3)	5 (5.8)	73 (83.9)	126.0 (109.4–146.6)	87 (33.1)
Xiantao Zhong Yi, ZYB-11, N95	12 (26)	58 (89.2)	6 (9.3)	1 (1.5)	15.1 (11.7–19.4)	65 (24.6)
Makrite, 9500, N95	6 (14)	47 (87.0)	7 (13.0)	-	12.4 (9.1–17.0)	54 (20.5)
3M, 9010, N95	5.5 (16)	7 (36.8)	5 (26.4)	7 (36.8)	44.9 (22.7–88.9)	19 (7.2)
3M, 9920H, PFF	18 (20)	6 (54.5)	2 (18.2)	3 (27.3)	34.1 (11.2–103.5)	11 (4.2)
Grande, CDN3S-P2, FFP2	12 (20)	9 (81.8)	-	2 (18.2)	13.8 (5.0–37.7)	11 (4.2)
PGT Care, PGT-0095, FFP2	3 (15)	4 (66.3)	1 (16.7)	1 (16.7)	21.0 (4.6–96.0)	6 (2.3)
Brand and model unknown, KN95 ^b^	6 (9)	6 (100)	-	-	2.8 (1.1–7.4)	6 (2.3)
Giko, 1200H, N95	6	1 (100)	-	-	-	1 (0.4)
Benehal, MS6115L, N95	3	-	1 (100)	-	-	1 (0.4)
Y&Z, Safety Work F720, N95	3	1 (100)	-	-	-	1 (0.4)
Lucca Light, Lucca Care, KN95/FFP2	-	1 (100)	-	-	-	1 (0.4)
Total	12 (18)	149 (56.6)	27 (10.3)	87 (33.1)	31.5 (26.3–37.8)	263

^a^ Median (interquartile range). ^b^ FFRs with no brand or model present on its surface.

## Data Availability

No new data were created or analyzed in this study. Data sharing is not applicable to this article.

## Supplementary Materials

The following supporting information can be downloaded at: https://www.mdpi.com/article/10.3390/ijerph20166618/s1, Table S1. Characteristics of 263 workers enrolled in the PROFIT study 2020. Table S2. Real-time and overall fit factor obtained in 16 FFRs excluded from the analysis. Table S3. Real-time and overall fit factor obtained in 263 FFRs included in the analysis.
