# Supplementary material for: Quantitative Fit Testing on Filtering Facepiece Respirators in Use by Peruvian Healthcare Workers Caring for Tuberculosis Patients during the COVID-19 Pandemic: PROFIT Study 2020
Source: Int J Environ Res Public Health. 2023 Aug 21;20(16):6618. doi: 10.3390/ijerph20166618 (PMC10454389; doi:10.3390/ijerph20166618)
Supplement: Supplementary file 1 [file ijerph-20-06618-s001.zip › ijerph-2470945-supplementary.pdf]

Table S1. Characteristics of 263 workers enrolled in the PROFIT study 2020.

	n (%)
Sex	
Female	219 (83.3)
Male	44 (16.7)
Age (median and interquartile range)	40 (RIC: 18)
Labor	
Nursing Assistant	72 (27.5)
Nurse	51 (19.5)
Doctor	36 (13.7)
Laboratory technician	23 (8.8)
Psychologist	15 (5.7)
Dentist	11 (4.2)
Nutritionist	9 (3.4)
Medical Technologist	9 (3.4)
Other's healthcare workers	23 (8.8)
Not reported	14 (5.4)
Working areas	
TB Control Program	89 (33.8)
Outpatient clinics and dental office	66 (25.1)
Triage and topic	27 (10.3)
Mycobacteriology laboratory	21 (8.0)
Radiology	6 (2.3)
Other's healthcare areas	32 (12.2)
Not reported	22 (8.4)

Table S2. Real-time and overall fit factor obtained in 16 FFR excluded from the analysis.

N	ID	Brand, Model, and Type of FF	Exclusion Criteria	FFR Usage Time (hours)	rt-FF Pre-instruction	rt-FF Post-instruction	Overall-FF
1	4118	3M, model 1860, type N95	verifiable FFR timeout [a]	16	7	6	5
2	6131	3M, model 1860, type N95	verifiable FFR timeout [a]	1	10	22	11
3	2230	3M, model 1860, type N95	high suspected counterfeit [b]	-	-	-	4
4	1102	3M, model 1860, type N95	high suspected counterfeit [c]	8	2	4	6
5	7131	3M, model 1860, type N95	high suspected counterfeit [c]	1	26	27	12
6	6216	Xiantao Zhong Yi, model ZYB - 11, type N95	facial hair	6	1	36	6
7	8226	Xiantao Zhong Yi, model ZYB - 11, type N95	facial hair	5	1	4	3
8	4218	3M, model 1860, type N95	facial hair	1	-	-	25
9	5232	3M, model 1860, type N95	facial hair	6	12	107	70

10	5121	3M, model 9010, type N95	facial hair	36	4	10	8
11	1121	Makrite, model 9500, type N95	facial hair	8	3	9	6
12	6125	Makrite, model 9500, type N95	facial hair	18	4	11	7
13	6135	Makrite, model 9500, type N95	facial hair	48	2	2	17
14	2128	GIKO, model 1200H, type N95	facial hair	3	20	20	15
15	4129	Grande, model CDN3S-P2, type FFP2	facial hair	12	2	2	3
16	6226	Xiantao Zhong Yi, model ZYB - 11, type N95	facial hair	4	2	9	3

[a] Small size

[b] Acquired on own account

[c] Provided by PHC

Table S3. Real-time and overall fit factor obtained in 263 FFR included in the analysis.

N	ID	Brand, Model, and Type of FF	rt-FF Pre-instruction	rt-FF Post-instruction	Overall-FF
1	1103	3M, model 1860, type N95	42	200	200
2	1107	Grande, model CDN3S-P2, type FFP2	38	200	168
3	1108	Makrite, model 9500, type N95	2	18	10
4	1110	Grande, model CDN3S-P2, type FFP2	3	11	20
5	1112	Makrite, model 9500, type N95	1	9	5
6	1114	3M, model 1860, type N95	Not done	Not done	173
7	1117	3M, model 1860, type N95	Not done	Not done	157
8	1118	3M, model 1860, type N95	19	200	172
9	1119	3M, model 1860, type N95	Not done	Not done	11
10	1120	3M, model 1860, type N95	38	76	10
11	1122	Xiantao Zhong Yi, model ZYB - 11, type N95	15	38	22
12	1123	Xiantao Zhong Yi, model ZYB - 11, type N95	Not done	Not done	9
13	1125	PGT Care, model PGT-0095, type FFP2	13	150	31
14	1127	3M, model 9010, type N95	42	72	13
15	1128	Xiantao Zhong Yi, model ZYB - 11, type N95	15	40	18

16	1129	3M, model 9010, type N95	52	200	123
17	1131	3M, model 1860, type N95	140	200	152
18	1134	Xiantao Zhong Yi, model ZYB - 11, type N95	15	45	38
19	1135	Makrite, model 9500, type N95	45	200	88
20	1201	3M, model 9920H, type PFF	3	10	6
21	1204	Makrite, model 9500, type N95	7	15	5
22	1205	3M, model 1860, type N95	13	200	109
23	1206	Xiantao Zhong Yi, model ZYB - 11, type N95	2	21	2
24	1207	3M, model 9920H, type PFF	5	11	10
25	1208	3M, model 9010, type N95	1	5	3
26	1209	Makrite, model 9500, type N95	5	44	6
27	1211	Makrite, model 9500, type N95	2	80	50
28	1212	Makrite, model 9500, type N95	2	5	4
29	1213	3M, model 1860, type N95	115	200	200
30	1215	3M, model 1860, type N95	Not done	Not done	200
31	1216	Xiantao Zhong Yi, model ZYB - 11, type N95	14	88	16
32	1218	3M, model 1860, type N95	Not done	Not done	13
33	1223	Makrite, model 9500, type N95	Not done	Not done	32
34	1224	3M, model 1860, type N95	Not done	Not done	131
35	1226	3M, model 1860, type N95	5	200	200
36	1230	Xiantao Zhong Yi, model ZYB - 11, type N95	Not done	Not done	27
37	1232	3M, model 1860, type N95	50	75	51
38	1233	Brand and model unknown, type KN95	Not done	Not done	6
39	1234	Xiantao Zhong Yi, model ZYB - 11, type N95	2	25	17
40	1236	Xiantao Zhong Yi, model ZYB - 11, type N95	14	33	21
41	2102	3M, model 1860, type N95	8	200	200
42	2103	3M, model 1860, type N95	200	200	200
43	2107	3M, model 9010, type N95	1	181	122

44	2108	3M, model 9010, type N95	1	200	108
45	2110	Makrite, model 9500, type N95	6	25	14
46	2112	PGT Care, model PGT-0095, type FFP2	4	20	6
47	2114	3M, model 1860, type N95	Not done	Not done	160
48	2117	Xiantao Zhong Yi, model ZYB - 11, type N95	Not done	Not done	17
49	2118	3M, model 1860, type N95	200	200	176
50	2119	3M, model 1860, type N95	Not done	Not done	154
51	2120	3M, model 1860, type N95	8	154	111
52	2121	Makrite, model 9500, type N95	22	50	46
53	2122	Xiantao Zhong Yi, model ZYB - 11, type N95	4	32	18
54	2123	Xiantao Zhong Yi, model ZYB - 11, type N95	Not done	Not done	38
55	2125	PGT Care, model PGT-0095, type FFP2	14	200	65
56	2127	Xiantao Zhong Yi, model ZYB - 11, type N95	52	70	51
57	2129	Makrite, model 9500, type N95	2	50	13
58	2131	3M, model 1860, type N95	200	200	200
59	2134	Xiantao Zhong Yi, model ZYB - 11, type N95	12	40	30
60	2135	Makrite, model 9500, type N95	13	35	27
61	2201	3M, model 9920H, type PFF	14	200	198
62	2204	Makrite, model 9500, type N95	5	70	28
63	2205	3M, model 1860, type N95	200	200	198
64	2207	Makrite, model 9500, type N95	1	50	3
65	2208	Brand and model unknown, type KN95	2	30	1
66	2209	Makrite, model 9500, type N95	2	105	43
67	2211	3M, model 1860, type N95	16	130	121
68	2212	Makrite, model 9500, type N95	2	5	8
69	2213	3M, model 1860, type N95	47	200	169
70	2215	3M, model 1860, type N95	Not done	Not done	200

71	2216	Xiantao Zhong Yi, model ZYB - 11, type N95	36	47	20
72	2218	3M, model 1860, type N95	Not done	Not done	169
73	2223	3M, model 9010, type N95	Not done	Not done	152
74	2224	3M, model 1860, type N95	Not done	Not done	127
75	2226	3M, model 9010, type N95	6	70	63
76	2232	3M, model 1860, type N95	8	4	25
77	2233	3M, model 1860, type N95	Not done	Not done	200
78	2234	Xiantao Zhong Yi, model ZYB - 11, type N95	2	4	2
79	2236	Brand and model unknown, type KN95	2	3	2
80	3102	Makrite, model 9500, type N95	2	40	80
81	3103	3M, model 1860, type N95	23	120	178
82	3107	3M, model 1860, type N95	93	200	148
83	3108	Brand and model unknown, type KN95	2	12	8
84	3110	3M, model 9010, type N95	90	200	181
85	3114	3M, model 1860, type N95	Not done	Not done	200
86	3117	Xiantao Zhong Yi, model ZYB - 11, type N95	Not done	Not done	12
87	3118	3M, model 1860, type N95	200	200	123
88	3119	3M, model 1860, type N95	Not done	Not done	71
89	3120	3M, model 1860, type N95	190	200	186
90	3121	3M, model 9010, type N95	60	200	79
91	3122	Xiantao Zhong Yi, model ZYB - 11, type N95	4	30	33
92	3123	Makrite, model 9500, type N95	Not done	Not done	26
93	3125	Makrite, model 9500, type N95	4	20	11
94	3127	3M, model 9010, type N95	7	38	34
95	3128	Makrite, model 9500, type N95	2	25	8
96	3129	Grande, model CDN3S-P2, type FFP2	4	110	42
97	3131	3M, model 1860, type N95	200	200	192
98	3135	Makrite, model 9500, type N95	31	70	24
99	3201	3M, model 9920H, type PFF	30	200	196

100	3204	Grande, model CDN3S-P2, type FFP2	12	50	19
101	3205	3M, model 1860, type N95	18	200	198
102	3206	Grande, model CDN3S-P2, type FFP2	11	60	5
103	3207	Brand and model unknown, type KN95	1	20	1
104	3209	Makrite, model 9500, type N95	3	40	11
105	3211	Makrite, model 9500, type N95	2	70	51
106	3212	Makrite, model 9500, type N95	1	8	7
107	3213	Xiantao Zhong Yi, model ZYB - 11, type N95	2	5	2
108	3215	3M, model 1860, type N95	Not done	Not done	120
109	3216	3M, model 1860, type N95	80	200	150
110	3218	3M, model 1860, type N95	Not done	Not done	152
111	3223	Makrite, model 9500, type N95	Not done	Not done	58
112	3224	3M, model 1860, type N95	Not done	Not done	198
113	3226	3M, model 1860, type N95	150	200	97
114	3230	Xiantao Zhong Yi, model ZYB - 11, type N95	Not done	Not done	3
115	3232	Xiantao Zhong Yi, model ZYB - 11, type N95	30	50	46
116	3233	3M, model 1860, type N95	Not done	Not done	174
117	3236	3M, model 1860, type N95	66	200	200
118	4102	Makrite, model 9500, type N95	1	20	14
119	4103	3M, model 1860, type N95	92	178	120
120	4107	Makrite, model 9500, type N95	20	30	35
121	4110	Makrite, model 9500, type N95	30	50	32
122	4114	3M, model 1860, type N95	Not done	Not done	23
123	4117	Xiantao Zhong Yi, model ZYB - 11, type N95	Not done	Not done	59
124	4119	3M, model 1860, type N95	Not done	Not done	71
125	4120	3M, model 1860, type N95	83	200	200
126	4121	Grande, model CDN3S-P2, type FFP2	9	45	4
127	4122	Xiantao Zhong Yi, model ZYB - 11, type N95	20	58	82

128	4123	Xiantao Zhong Yi, model ZYB - 11, type N95	Not done	Not done	24
129	4125	PGT Care, model PGT-0095, type FFP2	26	200	176
130	4127	Xiantao Zhong Yi, model ZYB - 11, type N95	5	28	32
131	4128	Makrite, model 9500, type N95	80	120	71
132	4131	3M, model 1860, type N95	65	200	121
133	4135	Makrite, model 9500, type N95	2	16	6
134	4201	Makrite, model 9500, type N95	2	7	3
135	4205	3M, model 1860, type N95	100	200	180
136	4206	Makrite, model 9500, type N95	2	21	2
137	4209	Grande, model CDN3S-P2, type FFP2	106	200	156
138	4211	3M, model 9010, type N95	2	100	16
139	4212	Makrite, model 9500, type N95	1	7	2
140	4213	3M, model 1860, type N95	200	200	172
141	4215	3M, model 1860, type N95	Not done	Not done	163
142	4216	3M, model 1860, type N95	100	200	200
143	4223	Benehal, model MS6115L, type N95	Not done	Not done	80
144	4224	3M, model 1860, type N95	Not done	Not done	163
145	4226	PGT Care, model PGT-0095, type FFP2	10	15	5
146	4230	Xiantao Zhong Yi, model ZYB - 11, type N95	Not done	Not done	41
147	4232	Xiantao Zhong Yi, model ZYB - 11, type N95	13	30	17
148	4233	3M, model 1860, type N95	Not done	Not done	27
149	4236	3M, model 9920H, type PFF	100	110	92
150	5102	Xiantao Zhong Yi, model ZYB - 11, type N95	2	4	3
151	5103	3M, model 1860, type N95	4	200	200
152	5114	3M, model 1860, type N95	Not done	Not done	148
153	5117	Xiantao Zhong Yi, model ZYB - 11, type N95	Not done	Not done	29
154	5118	Brand and model unknown, type KN95	15	22	5

155	5119	3M, model 1860, type N95	Not done	Not done	28
156	5120	3M, model 1860, type N95	200	200	139
157	5122	Xiantao Zhong Yi, model ZYB - 11, type N95	4	87	59
158	5123	Makrite, model 9500, type N95	Not done	Not done	9
159	5125	Makrite, model 9500, type N95	20	32	18
160	5127	3M, model 9010, type N95	15	200	156
161	5128	Xiantao Zhong Yi, model ZYB - 11, type N95	18	35	14
162	5131	3M, model 1860, type N95	60	200	198
163	5135	Makrite, model 9500, type N95	2	125	73
164	5201	Xiantao Zhong Yi, model ZYB - 11, type N95	15	60	34
165	5205	3M, model 1860, type N95	101	200	143
166	5209	Makrite, model 9500, type N95	11	55	34
167	5211	3M, model 9010, type N95	10	40	16
168	5215	3M, model 1860, type N95	Not done	Not done	173
169	5216	3M, model 1860, type N95	50	150	200
170	5218	Xiantao Zhong Yi, model ZYB - 11, type N95	Not done	Not done	16
171	5223	Xiantao Zhong Yi, model ZYB - 11, type N95	Not done	Not done	18
172	5224	3M, model 1860, type N95	Not done	Not done	165
173	5226	PGT Care, model PGT-0095, type FFP2	1	13	8
174	5230	Xiantao Zhong Yi, model ZYB - 11, type N95	Not done	Not done	16
175	5233	3M, model 1860, type N95	Not done	Not done	195
176	5236	3M, model 9920H, type PFF	9	21	2
177	6102	Makrite, model 9500, type N95	2	20	5
178	6103	3M, model 1860, type N95	Not done	Not done	49
179	6117	Xiantao Zhong Yi, model ZYB - 11, type N95	Not done	Not done	13
180	6118	3M, model 9010, type N95	3	95	52
181	6119	3M, model 1860, type N95	Not done	Not done	153

182	6120	Xiantao Zhong Yi, model ZYB - 11, type N95	2	2	2
183	6121	Y&Z, model Safety Work F720, type N95	1	1	1
184	6122	3M, model 9010, type N95	4	200	84
185	6123	Xiantao Zhong Yi, model ZYB - 11, type N95	Not done	Not done	24
186	6127	3M, model 9010, type N95	1	150	93
187	6128	Makrite, model 9500, type N95	8	40	15
188	6129	Makrite, model 9500, type N95	2	16	4
189	6201	3M, model 9920H, type PFF	10	100	49
190	6205	3M, model 1860, type N95	4	200	113
191	6211	3M, model 9010, type N95	2	50	1
192	6215	3M, model 1860, type N95	Not done	Not done	200
193	6218	Xiantao Zhong Yi, model ZYB - 11, type N95	Not done	Not done	4
194	6223	Xiantao Zhong Yi, model ZYB - 11, type N95	Not done	Not done	11
195	6224	3M, model 1860, type N95	Not done	Not done	200
196	6230	Xiantao Zhong Yi, model ZYB - 11, type N95	Not done	Not done	17
197	6232	Xiantao Zhong Yi, model ZYB - 11, type N95	30	60	36
198	6233	Xiantao Zhong Yi, model ZYB - 11, type N95	Not done	Not done	32
199	7102	Makrite, model 9500, type N95	1	3	1
200	7103	3M, model 1860, type N95	70	200	40
201	7117	Xiantao Zhong Yi, model ZYB - 11, type N95	Not done	Not done	187
202	7118	3M, model 9010, type N95	200	200	146
203	7120	Xiantao Zhong Yi, model ZYB - 11, type N95	20	50	14
204	7121	Makrite, model 9500, type N95	2	15	9
205	7122	Xiantao Zhong Yi, model ZYB - 11, type N95	20	168	77
206	7123	Makrite, model 9500, type N95	Not done	Not done	35

207	7127	Xiantao Zhong Yi, model ZYB - 11, type N95	6	40	8
208	7128	Makrite, model 9500, type N95	8	15	25
209	7129	Lucca Light, Lucca Care, KN95 / FFP2	1	4	4
210	7135	Makrite, model 9500, type N95	7	40	9
211	7201	Makrite, model 9500, type N95	9	25	6
212	7205	3M, model 1860, type N95	200	200	128
213	7209	Grande, model CDN3S-P2, type FFP2	3	6	4
214	7215	3M, model 1860, type N95	Not done	Not done	200
215	7216	Xiantao Zhong Yi, model ZYB - 11, type N95	5	35	15
216	7218	Xiantao Zhong Yi, model ZYB - 11, type N95	Not done	Not done	14
217	7223	Makrite, model 9500, type N95	Not done	Not done	4
218	7224	3M, model 1860, type N95	Not done	Not done	197
219	7226	Xiantao Zhong Yi, model ZYB - 11, type N95	2	25	7
220	7230	Xiantao Zhong Yi, model ZYB - 11, type N95	Not done	Not done	8
221	7232	Xiantao Zhong Yi, model ZYB - 11, type N95	50	70	31
222	7233	3M, model 1860, type N95	Not done	Not done	55
223	8102	Grande, model CDN3S-P2, type FFP2	1	2	5
224	8103	3M, model 1860, type N95	3	200	103
225	8117	Xiantao Zhong Yi, model ZYB - 11, type N95	Not done	Not done	11
226	8121	Xiantao Zhong Yi, model ZYB - 11, type N95	4	14	5
227	8122	Makrite, model 9500, type N95	16	14	41
228	8123	Makrite, model 9500, type N95	Not done	Not done	3
229	8128	Xiantao Zhong Yi, model ZYB - 11, type N95	3	10	4
230	8129	Xiantao Zhong Yi, model ZYB - 11, type N95	10	15	16
231	8201	3M, model 9920H, type PFF	9	18	5
232	8205	3M, model 1860, type N95	170	200	200
233	8209	Makrite, model 9500, type N95	9	20	18

234	8216	3M, model 1860, type N95	3	120	111
235	8218	Xiantao Zhong Yi, model ZYB - 11, type N95	Not done	Not done	4
236	8223	Xiantao Zhong Yi, model ZYB - 11, type N95	Not done	Not done	13
237	8224	3M, model 1860, type N95	Not done	Not done	197
238	8230	Xiantao Zhong Yi, model ZYB - 11, type N95	Not done	Not done	14
239	8233	3M, model 1860, type N95	Not done	Not done	120
240	9102	Makrite, model 9500, type N95	1	1	1
241	9117	3M, model 1860, type N95	Not done	Not done	200
242	9122	Xiantao Zhong Yi, model ZYB - 11, type N95	2	20	4
243	9123	Xiantao Zhong Yi, model ZYB - 11, type N95	Not done	Not done	2
244	9129	Makrite, model 9500, type N95	2	12	5
245	9201	3M, model 9920H, type PFF	4	75	45
246	9205	3M, model 1860, type N95	110	200	73
247	9209	3M, model 9010, type N95	4	110	36
248	9224	3M, model 1860, type N95	Not done	Not done	155
249	9230	Xiantao Zhong Yi, model ZYB - 11, type N95	Not done	Not done	7
250	9233	3M, model 1860, type N95	Not done	Not done	200
251	10102	Makrite, model 9500, type N95	2	8	5
252	10117	Xiantao Zhong Yi, model ZYB - 11, type N95	Not done	Not done	50
253	10122	Xiantao Zhong Yi, model ZYB - 11, type N95	6	60	22
254	10129	Grande, model CDN3S-P2, type FFP2	5	11	10
255	10201	3M, model 9920H, type PFF	50	200	77
256	10205	3M, model 1860, type N95	3	200	143
257	10209	Xiantao Zhong Yi, model ZYB - 11, type N95	8	15	9

258	11102	GIKO, model 1200H, type N95	8	25	32
259	11201	3M, model 9920H, type PFF	14	200	198
260	11205	3M, model 1860, type N95	3	200	168
261	11209	Makrite, model 9500, type N95	3	70	27
262	12102	Grande, model CDN3S-P2, type FFP2	1	1	2
263	12205	3M, model 1860, type N95	5	200	200
